# Effectiveness and Efficiency of Non-drug Therapy Among Community-Dwelling Adults With Hypertension in China: A Protocol for Network Meta-Analysis and Cost-Effectiveness Analysis

**DOI:** 10.3389/fmed.2021.651559

**Published:** 2021-02-25

**Authors:** Taihang Shao, Xia Li, Chengchao Zhou, Xiao Zang, Daniel C. Malone, Liang Zhang, Jifang Zhou, Wenxi Tang

**Affiliations:** ^1^Department of Pharmacoeconomics, School of International Pharmaceutical Business, China Pharmaceutical University, Nanjing, China; ^2^Center for Pharmacoeconomics and Outcomes Research, China Pharmaceutical University, Nanjing, China; ^3^Centre for Health Management and Policy Research, School of Public Health, Cheeloo College of Medicine, Shandong University, Jinan, China; ^4^Department of Epidemiology, School of Public Health, Brown University, Providence, RI, United States; ^5^Department of Pharmacotherapy, College of Pharmacy, University of Utah, Salt Lake City, UT, United States; ^6^School of Medical and Health Management, Tongji Medical College of Huazhong University of Science and Technology, Wuhan, China; ^7^Department of Public Affairs Management, School of International Pharmaceutical Business, China Pharmaceutical University, Nanjing, China

**Keywords:** non-pharmacological interventions, pharmacological interventions, community-based chronic disease management, hypertension, network meta-analysis, cost-effectiveness, China

## Abstract

**Introduction:** The Chinese government has established a nationwide community-based chronic disease management program since 2009 with hypertension a vital part of it. Though drugs have been proven effective with hypertensive patients, they bring economic burden as well, especially for those who with elevated blood pressure and are potentially eligible for national programs. When the effectiveness of pharmacotherapy-only interventions remains uncertain on these patients, non-pharmacological interventions have demonstrated non-inferior effectiveness and may have economic advantages. To date, there rarely are evidences on the effectiveness and cost-effectiveness of non-pharmacological treatment in comparison with pharmacological interventions for patients with varying severity of blood pressure. This study aims to propose a study for a network meta-analysis and cost-effectiveness analysis to explore what kind of intervention is potentially effective and cost-effective to four specific patient groups, stage I-III hypertensive patients and patients with elevated blood pressure, and to provide recommendations for hypertensive management to Chinese decision makers.

**Methods:** We will systematically search databases (MEDLINE, PubMed, Cochrane Library, etc.,) for randomized controlled trials and observational studies with qualified study design in recent decade that assess the effectiveness of non-pharmacological, pharmacological, or combined intervention aimed at adult populations who are diagnosed with the above four types of hypertension in China. The effectiveness outcomes will include changes in SBP/DBP, rate of comorbidities, mortality, and health related quality of life. We will use network meta-analysis to compare and rank effectiveness of different interventions. Subgroup analyses and meta-regression analyses will be performed to analyze and explain heterogeneity. The economic outcome will include cost-effectiveness based on simulation results from Markov models. Under study perspective of Chinese health system, life-time direct cost will be included.

**Discussion:** This study aims to compare and rank the effectiveness and cost-effectiveness of pharmacological, non-pharmacological and combined interventions for stage I–III hypertensive patients and those who with elevated blood pressure. Compared to existing studies, this comprehensive synthesis of relevant evidences will influence future practice with better efficiency and generalizability for community-based hypertensive management programs in China. The study might also be valuable for other low- and middle-income countries to find their own solutions.

**PROSPERO registration number:** CRD42020151518

## Introduction

Hypertension has a prevalence of 27.9% in residents over the age of 18 years (1) and has affected 270 million people in China alone until 2019 (2). Hypertension is one of the most common chronic diseases of the 21st century. Hypertension is also the most important cardiovascular risk factor and the leading cause of death in China (2). Research from the Chinese Center for Disease Control and Prevention (3) showed that in 2017, the number of people who expired due to hypertension-related diseases in China was 2.54 million, with approximately 69% died from stroke, 54% died from ischemic heart disease, and 41% died owing to other types of cardiovascular disease (CVD). The World Bank estimated that a 1% reduction in annual CVD events caused by hypertension between 2010 and 2040 could save China 10.7 trillion USD (4). According to the latest clinical practice guidelines for hypertension promulgated by the American College of Cardiology (ACC) in 2017, the diagnostic criterion for hypertension has dropped from 140/90 to 130/80 mmHg. This new standard may result in many more patients in China being diagnosed with hypertension (5). With accelerated population aging, together with lifestyle changes in modern society, elevated blood pressure (130–139/80–89 mmHg) and its consequences are too significant to ignore, clinically, economically, and humanistically. According to a study in the United States, 13.7% of American adults had blood pressure levels 130–139/80–89 mmHg, with 1.9% requiring antihypertensive medication (6). Current pharmacological interventions for hypertension are often ineffective and costly in controlling blood pressure, especially when they do not eradicate CVD events due to hypertension. Between 2005 and 2010, even with nearly 82% of American adults with hypertension aware of their status, and ~75% taking antihypertensive drugs, only 47% of patients with hypertension have uncontrolled blood pressure (7–9). The disease burden is also an unignorable factor. Another study on American population in 2015 showed that although the estimated per person costs associated with treating hypertension alone were 687 USD, the total medical costs with hypertension were much higher (5,458–6,038 USD), of which the cost related to CVD events was 1,067–1,156 USD (10). Furthermore, the widespread use of pharmacological interventions will also cause a medical burden on patients with elevated blood pressure.

Compared with pharmacological interventions, non-pharmacological interventions are community- or, population-based and have potential economic advantages. Currently, effective non-pharmacological interventions may include self-monitoring or self-management (11, 12), physician or pharmacist interventions (13, 14), and lifestyle or behavioral interventions (15, 16). In a study by Tucker et al. (11), self-monitoring was associated with reduced clinical systolic blood pressure (SBP), as compared with usual care at 12 months (−3.2 mmHg, 95% confidence interval [CI]: −4.9 to −1.6]). In a study by Santschi (13), compared with usual care, pharmacist interventions showed a greater reduction in SBP (7.6 mm Hg, 95% CI: 9.0–6.3) and diastolic blood pressure (DBP; 3.9 mm Hg, 95% CI: 5.1–2.8). Williamson et al. (16) found that at 3–6 months, exercise was associated with a reduction in SBP (−4.40 mmHg, 95% CI: −5.78 to −3.01) and in DBP (−4.17 mmHg, 95% CI: −5.42 to −2.93). In addition, according to these studies, the majority of cost of most non-pharmacological interventions was related to labor and overhead spending, attributes that can be easily reduced. A study in Nepal (17) showed that a community-based hypertension management program, including blood pressure monitoring, and lifestyle counseling, vs. usual care achieved an incremental cost-effectiveness ratio (ICER) of 582 USD per disability-adjusted life-year (DALY) averted, demonstrating it to be a highly cost-effective strategy.

Starting in 2009, China launched a reform of its primary health care system and established a basic public health service system focusing on chronic disease management (18). Hypertension is one of the main targets of the system. According to the “2017 National Basic Public Health Service Project Relevant Work and Requirements (19),” the per-capita expenditure for patients with hypertension is 1.35 USD per year. Specific projects include inspection, follow-up evaluation (follow-up frequency at least four times a year), and health examination. One study found that from 2009 to 2016, the Chinese government enacted 53 policies and 24 targeted chronic disease management in community health care institutions (20). These policies have achieved great changes in terms of enhancing primary care, community-based facilities were built and talents were trained (21, 22). Despite new policies being implemented in China, pharmacological interventions have not achieved the expected effects. According to one study in a Chinese population, 30.1% of all participants had taken prescription antihypertensive drugs but only 7.2% achieved control (23, 24). Currently in China, individuals with blood pressure ≥140/90 mmHg (25) are defined as being diagnosed with hypertension and are eligible for disease management. However, on the basis of a study in 2018, 46.4% of the Chinese adult population age ≥18 years may be categorized as hypertension using the new criterion of 130/80 mmHg. Regarding this, the application prospects of non-pharmaceutical interventions are enormous, and the need for related evidence to inform public health policies is urgent. However, current systematic reviews and meta-analyses in this area have evaluated one of the following three conditions: (1) conventional drug intervention vs. one kind of non-pharmaceutical intervention (14); (2) one particular drug vs. another (26, 27); (3) one particular kind of non-pharmaceutical intervention vs. usual care (11, 15, 16, 28, 29). There is lack of evidence in comparing all relative non-pharmaceutical interventions.

In this study, we aim to explore the types of intervention that are potentially effective as well as cost-effective for patients with stage 1, 2, or 3 hypertension and patients with elevated blood pressure (which has not been captured in stage 1 in China yet). The specific objectives of this study are as follows: (1) to identify and categorize currently available community-based non-pharmacological hypertension interventions, their applicability in various settings, and their specific measures using a comprehensive search protocol; (2) to evaluate the quality of the available evidence, the combined effectiveness of the interventions, and their ranking, and the reliability of the studies (e.g., heterogeneity, publication bias); (3) to explore potential subgroups (e.g., age, sex, and baseline activities of daily living [ADL]); (4) to develop an economic evaluation model of hypertension, with economic evaluation and ranking of costs and other parameters, by conducting a literature search and survey; (5) to analyze the appropriateness of the interventions and their generalizability in a community-dwelling population with hypertension; to provide a reference for the reform of chronic disease management among Chinese community-dwelling adults with hypertension.

## Materials and Methods

### Study Registration

This study will be conducted and reported under the guidance of the Preferred Reporting Items for Systematic review and Meta-Analysis Protocols (PRISMA-P) checklist (30) and Consolidated Health Economic Evaluation Reporting Standards (CHEERS) Statement (31). The protocol has been registered in the International Prospective Register of Systematic Reviews (PROSPERO) with registration number is CRD42020151518.

### Synthesis of Effectiveness

#### Inclusion Criteria for Study Selection

Study eligibility criteria are defined according to the PICOS (participants, interventions, comparisons, outcomes, study design) strategy.

##### Types of Study Designs

For the network meta-analysis, randomized controlled trials (RCTs) and clustered randomized controlled trial (CRCTs) will be included. In addition, quasi-experiments/natural experiments, case-control studies, cohort studies, and other observational studies with pre- and post-test designs will also be included to the extent the data are sufficient for inclusion. Given that this project will focus on community-based or population-based studies, Phase III clinical pharmacology trials will be excluded. Articles will be limited to those published in Chinese and English language within the past 10 years.

##### Participants

Participants should be Chinese adults >18 years old living in the community. Additionally, they should meet the requirement for one of four specific types of hypertension. The diagnostic criteria are as follows [according to the 2018 Chinese Guidelines for the Management of Hypertension (25)]: (1) patients with elevated blood pressure: 130–139/80–89 mmHg; (2) stage 1 hypertensive patients: 140–159/90–99 mmHg; (3) stage 2 hypertensive patients: 160–179/100–109 mmHg; (4) stage 3 hypertensive patients: ≥180/≥110 mmHg.

Community-dwelling patients with hypertension receiving non-pharmacological treatment, conventional drug intervention, or a combination of the two will be included, regardless of sex or age. According to the new standard of the ACC (5), patients with blood pressure 130–139/80–89 mmHg will also be included among the participants with elevated blood pressure. Community-dwelling patients with hypertension are defined as those who have been living in the community for more than 6 months, are conscious, and are willing to participate in the study (32). Patients with complications (e.g., diabetes mellitus or CVD) will be included, but those with hypertension owing to other causes (secondary hypertension, e.g., pregnancy or medication use) and pulmonary hypertension will be excluded.

We are concerned about the impact of non-pharmacological interventions in people with different levels of hypertension, and therefore we will divide patients into four groups with different stages of hypertension (stage 1, 2, or 3 hypertension and patients with blood pressure 130–139/80–89 mmHg) at the beginning of the study, for subsequent analysis.

##### Subgroup Analysis

The large heterogeneity of the population will lead to differences in the effectiveness among interventions, and therefore subgroups may also be included in the analysis, including age (young, middle-aged, older), sex, baseline ADL, presence of complications (e.g., diabetes, CVD events including Stroke, Coronary Heart Disease and Acute Myocardial Infarction), urban vs. rural residence, and follow-up time. Other potential subgroups will be included if data are available (employed vs. unemployed, insured vs. uninsured).

##### Types of Intervention/Comparator or Control Group

The included studies should address at least one type of condition: (1) non-pharmacological intervention vs. conventional drug intervention;(2) non-pharmacological intervention vs. control of usual care; (3) non-pharmacological intervention plus conventional drug intervention vs. conventional drug intervention; (4) other conditions that may be included in the study.

***Conventional drug intervention***: The scope of conventional drug intervention is set to include commonly used antihypertensive drugs recommended in the “China Hypertension Prevention and Control Guidelines 2018 Revised Edition (25),” including calcium channel blockers, angiotensin-converting enzyme inhibitors, angiotensin receptor antagonists, diuretics, and β-blockers.

***Non-pharmacological intervention***: Considering a broader range of non-pharmacological interventions, we intend to include evidence from studies evaluating the following interventions: patient-level interventions including: lifestyle; psychological interventions; social support; peer education; and self-management; and provider-level interventions including: human resources; service delivery; information technologies; and change management (see Table 1). The specific interventions will be acquired using search strategies and will be categorized by the researchers. We will also invite relevant public health experts to advise and verify the types of intervention. Studies that involve the use of multiple interventions will be included in the “combined intervention” and categorized into subgroups.

**Table 1:** Classification of interventions.

**Type of intervention**	**Intervention measures**
1. Service provision	1. Family health worker 2. Health education OR health education 3. Pharmaceutical care OR medication guidance OR pharmacist 4. Family doctor OR community doctor OR general practitioner
2. Service delivery method	1. Community-based OR joint management OR chronic disease management OR health management OR follow-up management OR individualized care 2. Quality of care OR evidence-based medicine OR continuity of care 3. Social support OR peer education 4. Service integration OR preventive medicine integration
3. Lifestyle intervention	1. Lifestyle 2. Patient behavior intervention 3. Sodium intake OR sodium restriction OR sodium reduction OR salt intake OR salt restriction OR salt reduction OR sodium chloride intake OR sodium chloride restriction OR sodium chloride reduction 4. Dietary approach OR diet 5. Weight loss OR weight reduction 6. Smoking cessation 7. Alcohol reduction OR alcohol restriction 8. Sports OR exercise OR physical activity OR physical fitness OR training OR tai chi OR yoga OR Baduanjin qigong OR dance
4. Psychological intervention	Psychological intervention
5. Medical information intervention	1. Telemedicine OR remote monitoring OR remote management 2. Online to offline 3. Internet hospital 4. Smart devices 5. Dynamic monitoring
6. Self-management	1. Self-management OR self-management group 2. Home blood pressure measurement OR self-test blood pressure
7. Chinese medicine	1. Traditional Chinese medicine 2. Acupuncture 3. Gua sha 4. Acupressure 5. Emotional therapy
8. Management transform	Medical union OR medical alliance OR integrated care delivery system
9. Comprehensive intervention	1. Comprehensive intervention measures OR multifaceted intervention OR intervention package 2. Combination of intervention types with at least two measures among the eight types above

*Specific search terms will be refined and described after article retrieval is completed*.

***Control***: The placebo (no-intervention) control groups will include routine care and management of hypertension, defined according to the 2018 Chinese Guidelines for the Management of Hypertension (25) and the National Standards for Basic Public Health Service (33) or no patient intervention.

It is important to note that articles included in the meta-analysis will also comprise economic evaluations involving cost accounting. Articles that describe interventions but do not specify expenditures for human and material resources will be excluded.

### Outcome Measures

#### Primary Outcomes

The primary outcomes include changes in SBP/DBP (or blood pressure control rate), CVD event rate, and mortality. The blood pressure control rate is defined as the proportion of community-dwelling patients with hypertension who have SBP <140 mmHg and DBP <90 mmHg (SBP <130 mmHg and DBP <80 mmHg for patients with complications) over a certain period of time (usually 3 months).

#### Secondary Outcome

The secondary outcome is health-related quality of life (quality-adjusted life-year [QALY] and health utility) assessed using validated scales, such as the EuroQol Five Dimensions questionnaire (EQ-5D) (34). Studies using non-utility scales such as the 36-Item Short-Form Health Survey (SF-36) (35) will only be included if the scores were mapped into health utility values. We will also capture QALY values if reported in an article.

### Search Strategies

The search will be conducted using the following databases or search platforms: PubMed, Embase, Cochrane Central Register of Controlled Trials (CENTRAL), the Cochrane Library, ClinicalTrials.gov, Chinese National Knowledge Infrastructure (CNKI), Chinese Scientific Journal Database (VIP), SinoMed, and Wanfang database.

Two rounds of searches will be conducted, reverse and forward. The reverse search will aim to identify intervention targets. The 2020 International Society of Hypertension global hypertension practice guidelines and the National Insurance Medicine List of China will be screened for drugs used in hypertension treatment; the Boolean operator NOT will be used to exclude studies using drugs in the intervention. The initial screening and a summary of the literature will be performed to identify and summarize non-pharmacological interventions with a high frequency of occurrence. The specific search strategy to be followed is described in Table 2.

**Table 2:** Reverse search strategy using PubMed database.

**No**.	**Search Items**
#1	Hypertension[Title/Abstract]
#2	(Controlled trial OR Quasi trial OR Case control OR Natural experiment OR Intervention)[Title/Abstract]
#3	Commonly used Western medicines (see Supplement Materials for details)
#4	Commonly used traditional Chinese medicines (see Supplement Materials for details)
#5	#3 AND #4
#6	(Influencing factors OR Mechanism)[Title]
#7	(Rat OR Pregnancy OR Mouse OR Animal OR Rabbit)[Title/Abstract]
#8	Community[Title/Abstract]
#9	China
#10	#1 AND #2 NOT #5 NOT #6 NOT #7 AND #8 AND #9

The forward search is aimed at including studies. We will use a combination of search strategies, developed using the interventions obtained in the previous step, to search and include the relevant literature. The goal of the two-stage search is to ensure the completeness and stability of the included literature. The specific search strategy is shown in Table 3.

**Table 3:** Forward search strategy using PubMed database.

**No**.	**Search items**
#1	Hypertension [Title/Abstract]
#2	(Controlled trial OR Quasi trial OR Case control OR Natural experiment OR Intervention) [Title/Abstract]
#3	Specific type of interventions (classified by the researchers)
#4	Review OR System Review OR Meta-Analysis [Title/Abstract]
#5	(Influencing factors OR Mechanism) [Title]
#6	(Rat OR Pregnancy OR Mouse OR Animal OR Rabbit) [Title/Abstract]
#7	#4 AND #5 AND #6
#8	Community [Title/Abstract]
#9	China
#10	#1 AND #2 AND #3 NOT #7 AND #8 AND #9

The studies involving pharmacoeconomic evaluations will be searched to collect relevant cost information, CVD event rates, and risk of death for non-pharmacological interventions, to identify relevant parameters in the economic model.

### Screening

All retrieved papers will be imported into NoteExpress (3.2.0.7535, User: China Pharmaceutical University), and two reviewers will read the title and abstract of all identified studies, to select all candidate papers. All duplicates will be eliminated. After title and abstract screening, full text eligible papers will be obtained for further evaluation. If one reviewer is unsure of the eligibility of an article, the full paper will be reviewed again. In the case of differing opinions between the two reviewers, a third reviewer, or other relevant authority will be consulted. All exclusions will be documented, along with the reasons for exclusion. The literature selection process will be illustrated in a flow diagram.

### Data Extraction

Two investigators will independently perform the data extraction. We will extract data of basic study characteristics: first author, year of publication, trial information (duration of the trial, registration information), study design (e.g., randomized controlled trial, cluster randomized controlled trial, quasi-experiment, case-control, and nested case-control), population (sample size, age, sex, diagnostic criteria for hypertension, health status, inclusion, and exclusion criteria), interventions (type and frequency of intervention, comparisons), outcomes (primary and secondary outcomes specified and collected, time points reported), setting, and risks of bias (sequence generation of the allocation; allocation concealment; blinding of participants, research personnel, and outcome assessors; incomplete outcome data; selective outcome reporting; and other sources of bias). Any disagreement in the data extraction will be resolved through discussion between two investigators, with further disagreement decided by a third investigator or other relevant authority. The distribution of patient baseline data will be determined based on the extracted data, and any literature that deviates from the distribution by a large amount and is clearly irrational will be excluded.

### Treatment of Missing Data

If there are any unreported data, we will attempt to contact the authors to obtain the missing information. If the data are still unable to be retrieved, we will include the study in the descriptive analysis, and the impact of missing data will be described in the discussion section.

### Risk of Bias Assessment

Two investigators will use relevant tools to evaluate the risk of bias for all included studies. For randomized controlled trials, we will use the Cochrane Risk of Bias Tool to evaluate the following six aspects: random sequence generation, allocation concealment, blinding of outcome assessors, completeness of outcome data, selective outcome reporting, and other potential bias. All six aspects will be evaluated as (1) low risk of bias, (2) unknown risk of bias, and (3) high risk of bias; a high-quality study should include more than four aspects with low risk of bias. However, given that the literature included in this study will mostly involve community-based trials and non-pharmacological interventions, blinding and allocation concealment will be difficult to achieve; therefore, we will make particular note of articles that do not involve blinding and allocation concealment but have valuable data.

### Assessment of Study Quality

For cohort studies, we will use the Newcastle-Ottawa (NOS) scale (36) to evaluate studies in terms of cohort selection, comparability between cohorts, outcome measures, and duration and completeness of follow-up. According to the NOS, a higher number of stars obtained after evaluation indicates higher quality of the study, up to a maximum of 10 stars. Generally, articles with at least 5 stars will be included in the meta-analysis.

We will use MINORS (methodological index for non-randomized studies) (37) to evaluate the quality of natural experiments. MINORS contains 12 evaluation items: (1) a stated aim of the study; (2) inclusion of consecutive patients; (3) prospective collection of data; (4) endpoints appropriate to the study aim; (5) unbiased evaluation of endpoints; (6) follow-up period appropriate to the main endpoints; (7) loss to follow up not exceeding 5%; (8) a control group receiving the gold standard intervention; (9) contemporary groups; (10) baseline equivalence of groups; (11) prospective calculation of the sample size; and (12) statistical analyses adapted to the study design. Each item is assigned a score of 0–2, with a maximum score of 24. The scoring is performed as follows: a score of 0 means not reported, 1 means reported but with insufficient information, and 2 means reported and sufficient information was provided.

### Quality of Evidence Assessment

We will evaluate the quality of evidence using the Grading of Recommendations, Assessment, Development, and Evaluations (GRADE) evidence rating approach (38), which classifies the evidence as high, medium, low, and very low quality. According to the GRADE method, we will measure the quality of evidence according to study limitations, imprecision, inconsistency, indirectness, and publication bias.

### Network Meta-Analysis

We will perform a network meta-analysis to compare the effectiveness of different non-pharmacological interventions in treating the community-dwelling population with hypertension, and we will rank these and their probabilities. Probability values will be reported using surface under the cumulative ranking curve (SUCRA) and ranked graphically using ranking plots. Both a network meta-analysis based on classical frequency statistics and a network meta-analysis based on the Bayes method will be conducted. Considering that the generalizability and effectiveness of an intervention at community level are not necessarily directly related, and that effectiveness is only one of the factors considered in the economic evaluation in the second phase of this study, a broad ranking will be considered; among the top 20 interventions, several will be selected for subsequent analysis in this study *via* consultation with experts and the researchers.

#### Effect Size

Risk difference (RD), hazard ratio (HR), and mean difference (MD) will be used for the effect sizes of count data and measurement data, and the risk and mean of each group will also be calculated to provide the basis for later decision tree modeling. Specifically, for SBP and DBP, the MD will be calculated using the confidence interval (CI) of its mean and standard deviation. If the CI is not reported in the literature, we will use the method recommended in the Cochrane Handbook, assuming the standard deviation (SD) correlation coefficient *r* = 0.5, and we will calculate the MD confidence interval based on the baseline and final follow-up SD data.

For the blood pressure control rate, the RR value will be used as an effect measure. For the CVD event rate, we will use the HR value as an index for survival data. Because a QALY is a continuous outcome, we will use the MD as the effect measure.

#### Assessment of the Transitivity Assumption

Standard methods to test transitivity are lacking, and therefore we will make subjective judgments about transitivity in terms of both clinical and methodological similarity. Clinical similarity includes the population, interventions, and follow-up time of each study, whereas methodological similarity refers to similarity in terms of study quality. Therefore, we will use a narrative approach to discuss the baseline characteristics of patients in the included studies, the definition and measurement of the outcome measures, design of combination therapy, follow-up time, and study practices.

#### Assessment of the Consistency Assumption

We will compare the consistency of evidence in head-to-head direct comparison and indirect comparison. We will use a node-splitting approach to assess the consistency between the two types of evidence. If there is no or little difference between the results of direct and indirect comparisons, the consistency hypothesis will be considered to be true. If inconsistencies occur, possible reasons for the inconsistencies will be explored in detail and an inconsistency model considered.

#### Dealing With Heterogeneity and Inconsistency

Heterogeneity among studies will be assessed by comparing the characteristics of patients, interventions, and trial designs, as well as the effect sizes and precision of outcomes, and will be finally assessed using statistical tests. Heterogeneity of the included outcomes will be analyzed using a χ^2^-test (test level = 0.05) combined with I^2^ quantification. Generally, I^2^ values >50% indicates heterogeneity, with values >70% suggesting significant differences across studies. If the statistical heterogeneity among the results is small(with I^2^ values smaller than 50%), the fixed-effects model will be used for meta-analysis; if the statistical heterogeneity among the results is large(with I^2^ values larger than 50%), the source of heterogeneity will be further analyzed and the random-effects model used for meta-analysis, after excluding the influence of obvious clinical or methodological heterogeneity. If there is obvious clinical or methodological heterogeneity, subgroup analysis and meta-regression will be used to explore the potential causes of heterogeneity and the impact on the combined effect size; alternatively, only qualitative description will be conducted. The test level for meta-analysis is set to α = 0.05. Meta-analysis will be performed using Stata SE 15 (StataCorp LLC, College Station, TX, USA).

### Publication Bias

If more than 10 studies are included, a funnel plot will be plotted to assess publication bias.

### Health Economic Modeling and Cost-Effectiveness Analysis

***Research perspective****:* A society-wide perspective will be applied, incorporating both direct and indirect costs.

**Figure 1:**
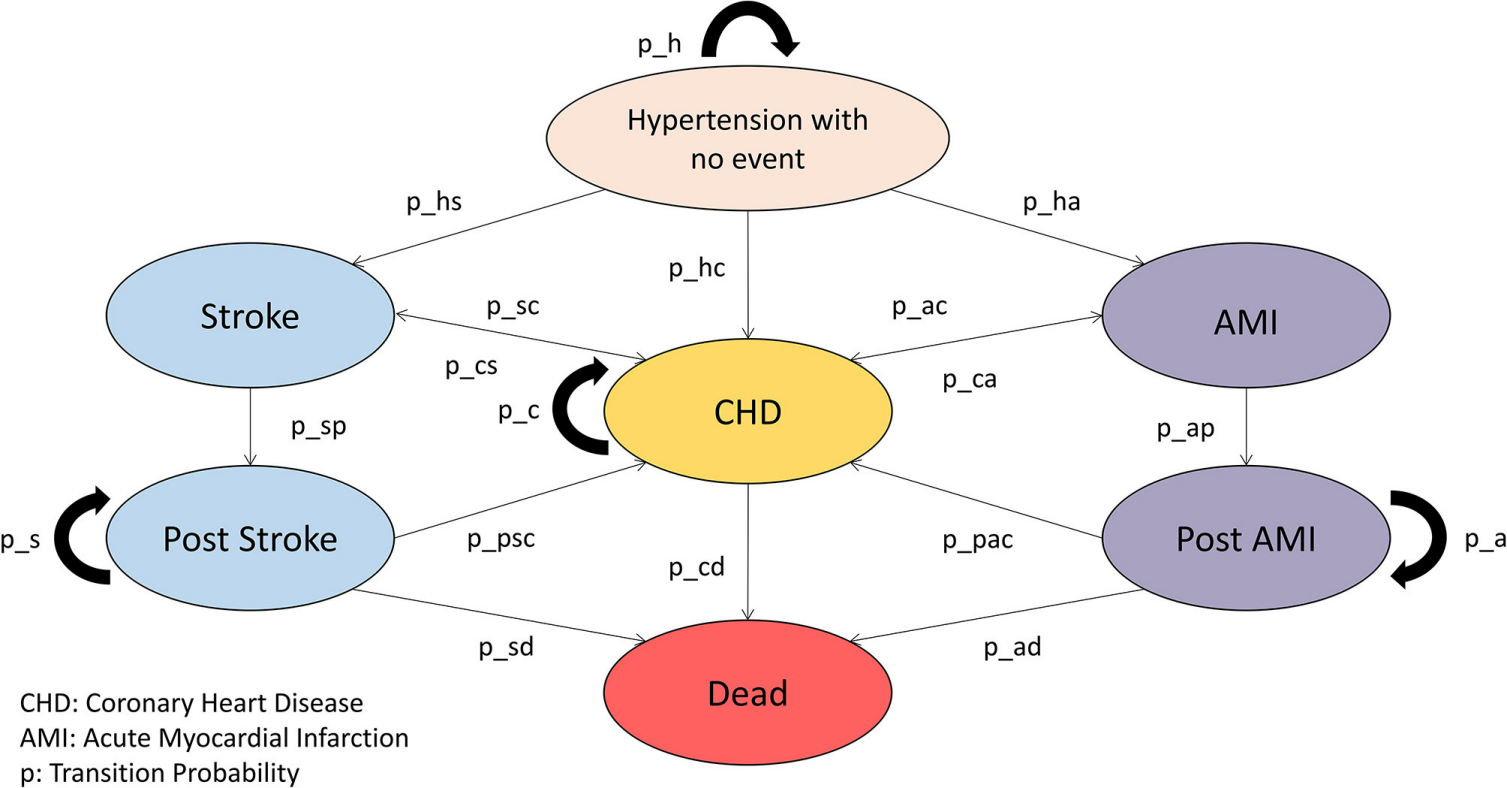
Markov model.

A Markov model will be established for the economic evaluation and an independent Markov model constructed for each intervention. The Markov model will be established, as shown in Figure 1. Health states of other comorbidities and complications as shown in Table 4 will also be considered in the Markov model.

**Table 4:** Health states in the Markov model.

**Category**	**Health states**
Comorbidities	Obesity, diabetes, dyslipidemia
Complications	Stroke, coronary heart disease, acute myocardial infarction, and heart failure

***Cycle****:* The cycle duration will be 3 months.

***Study duration****:* The study duration is the patient lifetime.

#### Economic Parameters

***Effectiveness****:* Effectiveness is represented by life-years, which can be simulated using the Markov model.

***Utility values****:* Utility values for each health state reported in the literature will be entered into the model; if these values were not reported in the literature, the utility data for each health state from an authoritative study of hypertension in China will be referenced, to ensure that the model works under this standard assumption.

***Transition probability****:* The incidence of CVD events reported in the literature for different study time frames is converted to a transition probability according to the model cycle.

$$\begin{array}{l} p=1-e^{-rt} \end{array}$$

where *p* is the probability of an event occurring within time *t* of the study mentioned in the article; *r* is the incidence of that event within time *t*, and *t* is the duration of the study (39).

***Natural mortality****:* Data of natural mortality will be obtained by consulting World Health Organization (WHO) data or the Chinese Statistical Yearbook.

***Disease mortality****:* Mortality data for each health state reported in the literature are to be entered into the model. If not reported in the literature, the mortality for each health state will be referenced from an authoritative Chinese study of hypertension to ensure comparability of model time frames under the standard assumptions.

***Cost standardization***: For costs, all resource consumption items will be standardized (minimum decomposition combined with human capital method to estimate the unit price), based on the description of the intervention in the included studies. The cost of each intervention is calculated by summing the item costs.

***Prices****:* Project prices reported in the literature will be discounted and entered into the model. If not reported in the literature, we will first obtain the validated intervention by combining the standardized operation in the guidelines or by consulting experts, and then weighting the price of drugs and medical services involved in the intervention by obtaining prices from the Chinese public pricing system.

***Discounting****:* We will discount the cost and effect outcomes to 2021 at a 5% discount rate to adjust for costs and outcomes over time. The rate we choose is in accordance with China Pharmacoeconomic Evaluation Guidelines 2019, and is higher than typical discount rates basically because of a faster growing GDP in China (40).

#### Economic Evaluation

***Decision indicator***: The ICER is calculated using the following formula:

$$\begin{array}{l} ICER=\frac{\Delta Cost\;of\;Intervention\;B\;than\;Intervention\;A}{\Delta QALYs\;of\;Intervention\;B\;than\;Intervention\;A} \end{array}$$

***Incremental analysis process****:* (1) eliminate any option with absolute dominance; (2) sort the alternative intervention measures in ascending order of cost; (3) calculate the incremental cost-effectiveness ratio of the intervention with minimal cost vs. zero-treatment and choose the one with cost-effectiveness (defined as ICER ≤ threshold of paying for one hypertensive patient effectively managed; (4) calculate the ICER of the second-smallest cost intervention with the remained option and run same decision; (5) make pairwise comparison in sequence until the final option is chosen.

***Threshold*:** China's 2019 GDP per capita (70,892 RMB) (41) will be used as the threshold criterion for cost-effectiveness. Because financial support for non-pharmacological interventions does not come from health insurance but rather from public health funds allocated in local budgets, a higher willingness to pay (e.g., twice the GDP per capita or lower) will be used as the threshold criterion.

### Dealing With Economic Uncertainty

#### Model Assumption

For the model structure, we will follow the economic model of hypertension adopted by the National Institute for Health and Care Excellence (NICE) (42–45), and the model will be validated by Chinese clinical experts.

#### Parameter Sensitivity Analysis

We will perform single-factor sensitivity analysis as well as probabilistic sensitivity analysis in the economic evaluation model. In the single-factor sensitivity analysis, deterministic sensitivity analysis (DSA), we will use the 95% CI of a single effect size as the fluctuation interval. For parameters lacking variance information, we assume that the relevant parameter fluctuates by 20–30% (considering the large cost uncertainty). Fluctuations in the discount rate range from 0 to 8%. The results of the DSA analysis will be presented in a tornado diagram. For probability sensitivity analysis (PSA), we will use a Monte Carlo simulation (10,000 iterations are expected). Prior distribution of the parameters will be applied, such as a beta distribution for transfer probability, effect value, and mortality, and gamma distribution for costs. The PSA results will be presented using a cost-effectiveness acceptability curve and incremental cost-effectiveness scatter plot.

## Discussion

Despite the increasing emphasis on community-based management of hypertension in China (46), compliance (47), affordability, and treatment rates of patients that rely on traditional pharmacological interventions must be improved. At present, a chronic disease management system based on non-pharmacological interventions has been established in Chinese communities; therefore, identifying community-appropriate interventions is of great importance for community health, representing an important direction for primary care and integrated health services moving forward and a challenge for policy makers. A variety of non-pharmacological interventions exist for hypertension, but comprehensive studies comparing their effectiveness and affordability are lacking. This study proposes a protocol for a network meta-analysis to synthesize all existing evidence and an economic evaluation on the cost-effectiveness of existing non-pharmacological hypertension interventions using an innovative and reliable method to classify different interventions. Study findings will provide implications for policymakers to identify the cost-effective alternative strategies, for local health systems to choose the best intervention package according to their actual resource levels, and provide community physicians and administrators with a basis for interventions. The methodology of this study will have implications for the management of other chronic diseases in the Chinese public health care system.

When classifying different groups of patients, we mainly rely on the 2018 Chinese Guidelines for the Management of Hypertension (25), which may miss some risk stratifications of in guidelines of other countries. When implementing this study, we will also discuss the influence of different guidelines [for example, ISH 2020 guidelines for HTA management and 2018 ESC/ESH guideline (48, 49)].

We hope that the results of our study would support evolutionary programs of chronic disease management nationwide, especially for population with risk factors but disease-free under current program inclusion criteria. We also hope that appropriate intervention strategies could be provided to precisely targeted patient groups based on comprehensive evidence of effectiveness, efficiency, and generalizability.

This study has several limitations. First, we expect there to be heterogeneity of interventions (including the “conventional” medication regimen in controls) across the papers that we identify. Second, because the trials will be community-based, the quality of the literature involved in this study will vary. Third, in constructing the economic model, the study may be biased because there is insufficient data arising from studies conducted in China. Therefore, some inputs may be obtained from jurisdictions outside of China. Fourth, we will address standardization of the cost approach. Finally, the literature included in this study will be in Chinese and English language only, which may exclude some relevant studies published in other languages conducted in the Chinese population; thus, language bias may be present.

## Author Contributions

WT conceptualized the research idea, developed the research design, and performed critical revision of this manuscript. TS drafted the manuscript. XL drafted and registered the protocol. JZ, XZ, CZ, DM, and LZ contributed to the study design. All authors read and approved the final manuscript.

## Conflict of Interest

The authors declare that the research was conducted in the absence of any commercial or financial relationships that could be construed as a potential conflict of interest.

## Abbreviations

CI: confidence interval
CVD: Cardiovascular disease
DBP: Diastolic blood pressure
ICER: Incremental cost effectiveness ratio
MD: Mean Difference
SBP: Systolic blood pressure.
