# Supplementary material for: Effectiveness and Efficiency of Non-drug Therapy Among Community-Dwelling Adults With Hypertension in China: A Protocol for Network Meta-Analysis and Cost-Effectiveness Analysis
Source: Front Med (Lausanne). 2021 Feb 25;8:651559. doi: 10.3389/fmed.2021.651559 (PMC7947298; doi:10.3389/fmed.2021.651559)
Supplement: Supplementary file 1 [file Table_1.DOCX]

**Supplement Materials**

| ***Name*** | ***Names of drugs*** |
| --- | --- |
| ***Commonly used Western medicines*** | Reserpine or Dibazol or Methyldopa or Clonidine or Prazosin or Ligustrazine or Ginkgo biloba or Ginkgo biloba extract or Ginkgo biloba honey ring or Dioscin or Compound Apocynum or Doxazosin or Naftopidil or Ula Diil or sodium nitroprusside or hydralazine or compound reserpine or compound reserpine or triamterene or hydrochlorothiazide or indapamide or furosemide or bumetanide or torasemide or triamterene or Spironolactone or Amiloride or Phentolamine or Sodium Ferulate or Dihydroergotine or Fasudil or Phenoxybenzamine or Pentoxifylline or Niergoline or Niacin or Inositol Nicotinate or Pancreatic kininogenase or diosmin or citrus flavone or compound carrageenate or heparin or polysulfonic mucopolysaccharide or aescin or troxerutin or propranolol or sotalol or atenolol Or bisoprolol or metoprolol or esmolol or arotinol or carvedilol or labetalol or amlodipine or nimodipine or nitrendipine or nifedipine or felodipine or amlodipine aspartate or amlodipine or atorvastatin or benidipine or lacidipine or lercanidipine or nicardipine or nicardipine or nicardipine or nicardipine or levondipine or levoamlodipine Dipine or diltiazem or verapamil or captopril or enalapril or benazepril or fosinopril or lisinopril or ramipril or imidapril or perindopril Li or Amlodipine Benazepril or Benazepril Hydrochlorothiazide or Compound Captopril or Lisinopril Hydrochlorothiazide or Enalapril Folic Acid or Perindopril Indapamide or Perindopril Amlodipine or Valsartan or Olmesartan medoxomil or Irbesartan or Losartan or Telmisartan or Candesartan medoxomil or Olmesartan medoxomil Hydrochlorothiazide or Irbesartan Hydrochlorothiazide or Losartan Hydrochlorothiazide or Telmisart Tan hydrochlorothiazide or valsartan amlodipine or valsartan hydrochlorothiazide or olmesartan medoxomil and amlodipine or telmisartan amlodipine or candihydrothiazide or manidipine or barnidipine or chlorthalidone or epri Ketone or betalol or Cilapril or Minoxidil or Aliskiren |
| ***Commonly used traditional Chinese medicines*** | Songling Xuemaikang Capsules or Niuhuang Antihypertensive Pills or Zhenju Antihypertensive Tablets or Tianma Gouteng Granules or Compound Apocynum Tablets or Xinkeshu Pills or Xinmaitong Tablets or Qinggan Jiangya Capsules or Annao Pills or Naoliqing pills or Qianglidingxuan tablets or Angong Jiangya pills or Xinxuening tablets or Camellia antihypertensive tablets or Tianshu tablets or Maijunan tablets or Yufengningxin pills or Duzhong granules or Xin'an capsules or Zhennaoning Capsule or Qingnao Antihypertensive Tablets or Mailuotong Granules or Seventy Flavor Pearl Pills or Twenty Five Flavor Coral Pills or Twenty Flavor Chenxiang Pills or Wulan Thirteen Flavor Soup Powder or Shan Paste Seventy Flavor Pills or Twenty Five Flavor Coral Capsule or Changsong Bawei Chenxiang Powder or Compound Gaozi Patch Tablets or Longdanxiegan Capsules or Niuhuang Qingxin Tablets or Dioscin Tablets or Quick-acting Niuhuang Pills or Soul Pills or Antihypertensive Avoiding Wind Tablets or Kangmaixin Oral Liquid or Wujin Oral Liquid or Xingnaoling Tablets or Tianma Shouwu Tablets or Yuntongding Tablets or Shuxinning Tablets or Xianling Spleen Tablets or Compound Duzhong Tablets or Xinnaojing Tablets or Duzhong Antihypertensive Tablets or Qingxuan Antihypertensive Tablets or Compound Tianma Granules or Awakening Nao Anshen Tablets or Nourishing Yin and Antihypertensive Capsules or Shuangdan Ointment or Xinnaojian Capsules or Thrombolytic Capsules or Twenty-five Flavors Yuganzi Pills or Thirteen Flavors Honghua Pills or Yinaoning Pills or Xingnao Antihypertensive Pills or Heart Shubao Capsules or Thirteen Flavors Maqian Pills or Hawthorn Antihypertensive Capsules or Baiai Capsules or Apocynum Antihypertensive Tablets or Apocynum venetum Leaf Capsules or Eucommia Antihypertensive Capsules or Eucommia Double Dropping Bags or Uncaria Cassia Granules or Kuding Antihypertensive Capsules or Blood Pressure Ambabu Ointment or Guanxinkang Tablets or Huixinkang Tablets or Duzhong Capsules or Hawthorn Extract Lipid-lowering Tablets or Xieqing Pills or Angelica Longyun Pills or Dizziness Ning Tablets or Xin'an Ning Tablets or Brain Liqing Capsules or Jiannao Bushen Pills or Yiling Jing or Guilu Bushen Capsules or Modified Zhenwu Decoction Traditional Chinese Medicine or Rougan Qingxuan Decoction or Blood Pressure and Lipid Reducing Capsules or Blood Pressure Capsules or Yishen Antihypertensive Recipe or Tianma Gouteng Drink or Herbs for suppressing the liver and suppressing Yang or Nourishing Liver Decoction or Nourishing the Kidney and Removing Phlegm and Removing Blood Stasis Decoction or Compound Maoma Capsules or Warming and Nourishing Kidney Yang Method or Pinggan Decoction or Yiqi Smart Decoction or Yiqi Huatan Decoction or Banxia Baizhu Tianma Decoction or Yiqi Shugan Huoxue Decoction or Bushen Yixin Tablets or Gastrodia Uncaria Decoction or Qingxuan Anti-hypertensive Decoction or Scutellaria Baicalensis or Salvia Miltiorrhiza or Uncaria or Borneol or Prunella or Gardenia or Gastrodia or Turmeric or Pueraria lobata or Safflower or Bezoar or Mother of Pearl or Cinnabar or Gan Grass or woody or pearl or chuanxiong or myrobalan or coptis or hawthorn or buffalo horn |
